# Nanofat Improves Vascularization and Tissue Integration of Dermal Substitutes without Affecting Their Biocompatibility

**DOI:** 10.3390/jfb15100294

**Published:** 2024-10-03

**Authors:** Francesca Bonomi, Ettore Limido, Andrea Weinzierl, Emmanuel Ampofo, Yves Harder, Michael D. Menger, Matthias W. Laschke

**Affiliations:** 1 Institute for Clinical and Experimental Surgery, Saarland University, 66421 Homburg, Germany; francescabonomi.bonnie@gmail.com (F.B.); limidoettore@gmail.com (E.L.); andrea.weinzierl@icloud.com (A.W.); emmanuel.ampofo@uks.eu (E.A.); michael.menger@uks.eu (M.D.M.); 2 Department of Surgery, Ospedale Beata Vergine Mendrisio, Ente Ospedaliero Cantonale (EOC), 6850 Mendrisio, Switzerland; 3 Department of Plastic Surgery and Hand Surgery, University Hospital Zurich, 8006 Zurich, Switzerland; 4 Department of Plastic, Reconstructive and Aesthetic Surgery and Hand Surgery, Centre Hospitalier Universitaire Vaudois (CHUV), 1005 Lausanne, Switzerland; yves.harder@yahoo.com; 5 Faculty of Biology and Medicine, University of Lausanne (UNIL), 1005 Lausanne, Switzerland

**Keywords:** skin regeneration, nanofat, dermal substitutes, Integra^®^, vascularization, angiogenesis, inflammation

## Abstract

Dermal substitutes require sufficient tissue integration and vascularization to be successfully covered with split-thickness skin grafts. To rapidly achieve this, we provide the proof of principle for a novel vascularization strategy with high translational potential. Nanofat was generated from subcutaneous adipose tissue of green fluorescence protein (GFP)^+^ C57BL/6J donor mice and seeded onto small samples (4 mm in diameter) of the clinically approved dermal substitute Integra^®^. These samples and non-seeded controls were then implanted into full-thickness skin defects in the dorsal skinfold chamber of C57BL/6J wild-type mice and analyzed by intravital fluorescence microscopy, histology and immunohistochemistry over a 14-day period. Nanofat-seeded dermal substitutes exhibited an accelerated vascularization, as indicated by a significantly higher functional microvessel density on days 10 and 14 when compared to controls. This was primarily caused by the reassembly of GFP^+^ microvascular fragments inside the nanofat into microvascular networks. The improved vascularization promoted integration of the implants into the surrounding host tissue, which finally exhibited an increased formation of a collagen-rich granulation tissue. There were no marked differences in the inflammatory host tissue reaction to nanofat-seeded and control implants. These findings demonstrate that nanofat significantly improves the in vivo performance of dermal substitutes without affecting their biocompatibility.

## 1. Introduction

The management of large, full-thickness skin defects caused by burn injuries, trauma, tumor resection or systemic conditions, such as diabetes, represents a major challenge in reconstructive surgery [1]. Split-thickness skin grafts (STSGs) are commonly used to reconstruct and cover such defects. However, a major prerequisite for their survival during the initial post-transplantation phase is a sufficient vascularization of the underlying host tissue. Furthermore, STSGs without dermal support are prone to scarring and contraction with functional and esthetic limitations [2]. To overcome these problems, dermal substitutes (acellular dermal matrices or biologic meshes) have been introduced as temporary scaffolds to support dermal tissue regeneration by providing a favorable environment for cell migration, proliferation and differentiation [3].

One widely used, clinically approved dermal substitute is Integra^®^ (Integra Life Sciences, Plainboro, NJ, USA), which consists of cross-linked bovine tendon collagen and a shark glycosaminoglycan matrix covered by a silicone sheet temporarily replacing the epidermal layer [4,5,6]. In clinical practice, Integra^®^ is directly implanted onto a clean wound bed. After sufficient integration into the surrounding tissue and vascularization, the silicone sheet is removed and the dermal substitute is covered with an STSG. A major drawback of this two-step approach is the time-consuming vascularization of the dermal substitute underlying physiological kinetics of angiogenesis. The growth of newly developing blood vessels proceeds at an average rate of 5 µm/h [7,8]. Hence, complete vascularization of the dermal substitute usually requires 4–6 weeks, depending on various factors including the wound milieu and the thickness of the matrix. During this critical period, patients are exposed to a risk of wound infections as long as the physiological barrier of the skin is not completely re-established.

Recent studies highlight the potential of autologous adipose-tissue-derived microvascular fragments (MVFs) as vascularization units for dermal substitutes [9,10]. When seeded onto Integra^®^, they rapidly interconnect with each other into new microvascular networks, markedly accelerating its vascularization and integration and, thus, its subsequent and successful coverage with STSGs [10]. However, MVFs are enzymatically isolated from fat tissue, which may not be easily implemented into clinical routine because of regulatory hurdles and difficulties in standardizing the isolation procedure due to dissimilar enzyme activities between different lots [11,12].

These challenges may be overcome by the use of nanofat, which can be rapidly generated intraoperatively by mechanical emulsification and filtration of fat samples without adding enzymes or other supplements. Accordingly, nanofat represents a random mixture of microvessel segments, adipose-derived stem cells (ADSCs), extracellular matrix components and biological peptides with a high growth factor content and regenerative capacity [13,14,15]. In the present study, we used this mixture of molecular and cellular components for the first time for the seeding of Integra^®^ to provide the proof of principle that nanofat is a potent autologous product accelerating and improving vascularization and tissue integration of dermal substitutes.

## 2. Materials and Methods

### 2.1. Animals

Dorsal skinfold chambers were implanted in 16 C57BL/6J wild-type mice (male and female; Institute for Clinical and Experimental Surgery, Saarland University, Homburg, Germany). They exhibited an age of ~5 months and a body weight of ~25 g. Nanofat was generated from the inguinal fat tissue of 8 green fluorescent protein (GFP)^+^ donor mice (C57BL/6-Tg (CAG-EGFP)131Osb/LeySopJ; The Jackson Laboratory, Bar Harbor, ME, USA). They exhibited an age of ~5 months and a body weight of ~30 g. Using these different mouse strains enabled the identification of GFP^+^ cells from the nanofat and GFP^-^ cells from the host tissue. The mice that were equipped with dorsal skinfold chambers were housed in individual cages at a temperature of 22–24 °C, a relative humidity of 50–60% and a 12 h light/dark cycle for the entire duration of the experiment. All animals were fed ad libitum with standard pellet chow (Altromin, Lage, Germany) and had free access to water.

### 2.2. Anesthesia

The harvesting of nanofat, the preparation of the dorsal skinfold chamber and the microscopic analyses were performed after anesthetizing the animals by an intraperitoneal injection (i.p.) of 100 mg/kg body weight ketamine hydrochloride (Ketabel^®^; Bela-pharm GmbH & Co. KG, Vechta, Germany) and 12 mg/kg body weight xylazine (Rompun^®^; Bayer, Leverkusen, Germany). Postoperative analgesia was achieved by a subcutaneous injection of 10 mg/kg body weight carprofen (Rimadyl^®^; Zoetis Deutschland GmbH, Berlin, Germany).

### 2.3. Generation of Nanofat

**Figure 1:**
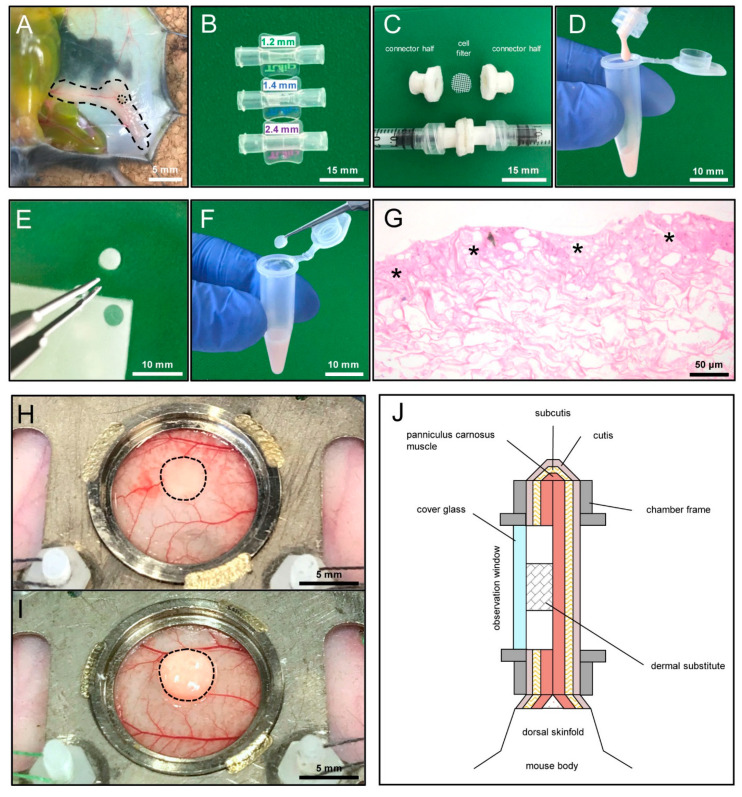
Methodological approach of this study. (**A**) Inguinal subcutaneous adipose tissue of a green fluorescence protein (GFP)^+^ donor mouse (border = broken line) with its inguinal lymph node (border = dotted line) for the generation of nanofat. (**B**) Three Luer-Lock connectors with descending internal diameters (2.4 mm, 1.4 mm, 1.2 mm) for mechanical fat emulsification. (**C**) Cell filter (pore size of 500 µm) sandwiched between two Luer-Lock connector halves for the filtration of the emulsified fat. (**D**) Freshly generated nanofat exhibiting a typical liquid consistency. (**E**) Sample that was cut out of a 1.3 mm thick, single-layer Integra^®^ dermal regeneration template without a silicone sheet by means of a 4 mm biopsy punch. (**F**) Incubation of the sample in freshly generated nanofat. (**G**) Hematoxylin–eosin (HE)-stained cross-section of the sample after the seeding process. The nanofat is mainly located on the surface of the sample (asterisks). (**H**,**I**) Observation window of the dorsal skinfold chamber after implantation of a non-seeded (control, (**H**)) and nanofat-seeded (**I**) dermal substitute (border = broken line). (**J**) Schematic cross-section of a dorsal skinfold chamber with an implanted dermal substitute.

Nanofat was generated according to standard procedures [15], applied under clinical conditions. Briefly, anesthetized GFP^+^ donor mice were sacrificed by means of cervical dislocation. The inguinal subcutaneous adipose tissue was then excised, avoiding the harvest of inguinal lymph nodes (Figure 1A). After washing in 0.9% NaCl, the tissue was minced using a tissue cutter (McIlwain Tissue Chopper; CLE Co., Ltd., Gomshall, UK). This resulted in fat fragments of ~1 × 1 × 1 mm. These fragments were subsequently emulsified by shuffling them 30 times between two syringes with Luer-Lock connectors of descending internal diameters (2.4 mm, 1.4 mm, 1.2 mm) (Figure 1B). The emulsified fat was finally passed through a cell filter (pore size: 500 μm) to remove larger tissue pieces (Figure 1C,D).

### 2.4. Seeding of Dermal Substitute with Nanofat

A dermal single-layer regeneration template without a silicone sheet (Integra^®^; Integra Life Sciences, Gent, Belgium) with a thickness of 1.3 mm served for the preparation of small implant samples by means of a 4 mm biopsy punch (Kai Europe GmbH, Solingen, Germany) (Figure 1E). These samples were then transferred into a tube (Eppendorf, Hamburg, Germany) filled with freshly generated liquid nanofat for 10 min (Figure 1F). This resulted in the coverage of the matrix surface with nanofat, as shown on histological sections of the samples taken to check the seeding efficiency (Figure 1G).

### 2.5. Dorsal Skinfold Chamber Model

The in vivo performance of nanofat-seeded and non-seeded dermal substitutes was analyzed in the mouse dorsal skinfold chamber model by means of intravital fluorescence microscopy, as described previously in detail [16]. C57BL/6J wild-type mice were anesthetized and their dorsal skin was first shaved and then chemically depilated (asid-med depilation cream; Asid Bonz GmbH, Herrenberg, Germany). Thereafter, two symmetrical low-weight titanium frames (Irola Industriekomponenten GmbH & Co. KG, Schonach, Germany) were fixed on their extended dorsal skinfold. One layer of skin with the panniculus carnosus muscle was removed in a circular preparation area with a diameter of ~15 mm. This area was used for microscopic analyses through the chamber observation window.

After the preparation of the dorsal skinfold chamber, the animals were allowed to recover for 48 h. Then, the observation window of the chamber was opened, to implant a non-seeded (control, *n* = 8) or nanofat-seeded (*n* = 8) dermal substitute in its center (Figure 1H,I). Afterward, the observation window was closed again by means of a removable cover glass and a snap ring (Figure 1J).

Throughout the entire time course of the dorsal skinfold chamber experiments, the general condition of the animals was monitored daily according to a standardized score sheet, which included the repeated assessment of cleaning and movement behavior, posture, body weight as well as potential injuries due to the implanted titanium frames. Of note, the animals tolerated the dorsal skinfold chambers well, as indicated by normal behavior and activity. They only exhibited a slight weight loss during the initial 2 days after the implantation of the chambers. Thereafter, they again showed an increasing body weight, indicating their rapid adaptation to the implants.

### 2.6. Stereomicroscopy and Intravital Fluorescence Microscopy

The dermal substitutes were repeatedly analyzed by means of stereomicroscopy on days 0 (day of implantation), 3, 6, 10 and 14 after fixation of the anesthetized mice on a Plexiglas^®^ stage. This allowed the horizontal positioning of the observation window of the dorsal skinfold chamber under a stereomicroscope (Leica M651, Wetzlar, Germany) that was connected with a camera and DVD recording system.

The vascularization of the implants was additionally analyzed in more detail using intravital fluorescence microscopy. For this, we injected a mixture of 0.05 mL of 5% fluorescein isothiocyanate (FITC)-labeled dextran (150,000 Da; Sigma-Aldrich, Taufkirchen, Germany) and 0.05 mL of 0.1% rhodamine 6G (Sigma-Aldrich) into the retrobulbar venous plexus of the anesthetized mice. These two fluorescent dyes served for the staining of blood plasma and leukocytes, respectively. The observation window of the chamber was horizontally placed under a Zeiss Axiotech fluorescence epi-illumination microscope (Zeiss, Oberkochen, Germany) that was equipped with a charge-coupled device camera (Kamara Axiocam 702 mono; Carl Zeiss Microscopy, Oberkochen, Germany) to transfer and store the microscopic images on a hard drive for later offline measurements by means of the analysis system CapImage (version 8.10.1; Zeintl, Heidelberg, Germany).

Implant vascularization was investigated in 8 regions of interest (ROIs) in the center (*n* = 4) or the border zones (*n* = 4) of the implants. Perfused ROIs (given in % of all ROIs) were defined as ROIs that exhibited newly formed red blood cell (RBC)-perfused microvessels. In addition, the functional microvessel density was measured as the length of all RBC-perfused microvessels per ROI (given in cm/cm^2^). Moreover, the diameter (d, given in µm) and centerline RBC velocity (v, given in µm/s) of 5 randomly selected microvessels per ROI were assessed. These microhemodynamic parameters were then used to calculate the wall shear rate (y, given in s^−1^) by means of the Newtonian definition y = 8 × v/d and the volumetric blood flow (vq, given in pL/s) vq = π × (d/2)^2^ × v/k where k = 1.6 represents the Baker–Wayland factor [17].

To assess the inflammatory response to the implants, microhemodynamic parameters (diameter, centerline RBC velocity, shear rate and volumetric blood flow) and leukocyte–endothelial cell interactions of postcapillary and collecting venules within the host tissue were assessed in 4 different ROIs in the direct vicinity of the implants. Leukocytes were assigned to free-flowing, rolling or adherent cells dependent on their interaction with the endothelium. Rolling leukocytes (given in min^−1^) exhibited a velocity less than two-fifths of the centerline velocity. Adherent leukocytes (given in mm^−2^ of endothelial surface) attached to the microvascular endothelium for at least 30 s. The endothelial surface was calculated from the diameter and length of the corresponding vessels based on the assumption that the vessels exhibited a cylindrical geometry.

### 2.7. Histology and Immunohistochemistry

At the end of the in vivo experiments, the mice were sacrificed by cervical dislocation under anesthesia. The chamber host tissue with the implants was carefully excised, fixed in 4% formalin and embedded in paraffin to cut 3 µm thick serial sections, which were stained with hematoxylin–eosin (HE). Additional sections were stained with primary antibodies against CD31 (1:100; dianova GmbH, Hamburg, Germany), lymphatic vessel endothelial hyaluronan receptor (LYVE)-1 (1:200; Abcam, Cambridge, UK) and GFP (1:200; Rockland, Limerik, PA, USA) as well as collagen (Col) I (1:250; Abcam), Col III (1:100; Proteintech, Rosemont, IL, USA), CD68 (1:300; Abcam), CD3 (1:100; Abcam) and myeloperoxidase (MPO) (1:100; Abcam). A goat-anti-rat IgG-Alexa555 antibody (1:100; Molecular Probes, Eugene, OR, USA), an anti-rabbit IgG-Alexa555 antibody (1:200; Molecular Probes), a donkey-anti-goat biotin-labeled antibody (1:50; dianova GmbH) with Alexa488-labeled streptavidin (1:50; Molecular Probes) and a biotinylated goat-anti-rabbit IgG antibody (ready-to-use; Abcam) with peroxidase-labeled streptavidin (ready-to-use; Abcam) and 3-amino-9-ethylcarbazole as chromogen (Abcam) served as secondary antibodies. Hoechst 33,342 (2 μg/mL; Sigma-Aldrich) was used for the staining of cell nuclei on immunofluorescence sections.

The quantitative analysis of the sections was performed with a BX53 microscope and the imaging software cellSens Dimension (version 1.11; Olympus, Hamburg, Germany). The density (given in mm^−2^) of CD31^+^ microvessels and LYVE-1^+^ lymph vessels was assessed in the center and border zones of each implant by dividing the number of all vessels by the analyzed tissue area. The fraction of GFP^+^ blood and lymph vessels (given in % of all vessels) was measured in the group of nanofat-seeded dermal substitutes. In addition, the total Col I and Col III ratio (Col content in implants in relation to normal skin) and the numbers of CD68^+^ macrophages (given in mm^−2^), CD3^+^ lymphocytes (given in mm^−2^) and MPO^+^ neutrophilic granulocytes (given in mm^−2^) were assessed in 2 ROIs in the border zones and 2 ROIs in the center of each implant.

### 2.8. Statistical Analysis

All data were first tested for a normal distribution and equal variance. Differences between two groups were assessed by an unpaired Student’s *t*-test (GraphPad Prism 10.1.2; GraphPad Software, San Diego, United States). A Mann–Whitney rank-sum test was used for non-parametric data. All values are shown as the mean ± standard error of the mean (SEM). Statistical significance was defined for a *p*-value < 0.05.

## 3. Results

### 3.1. In Vivo Microscopy of Dermal Substitutes

**Figure 2:**
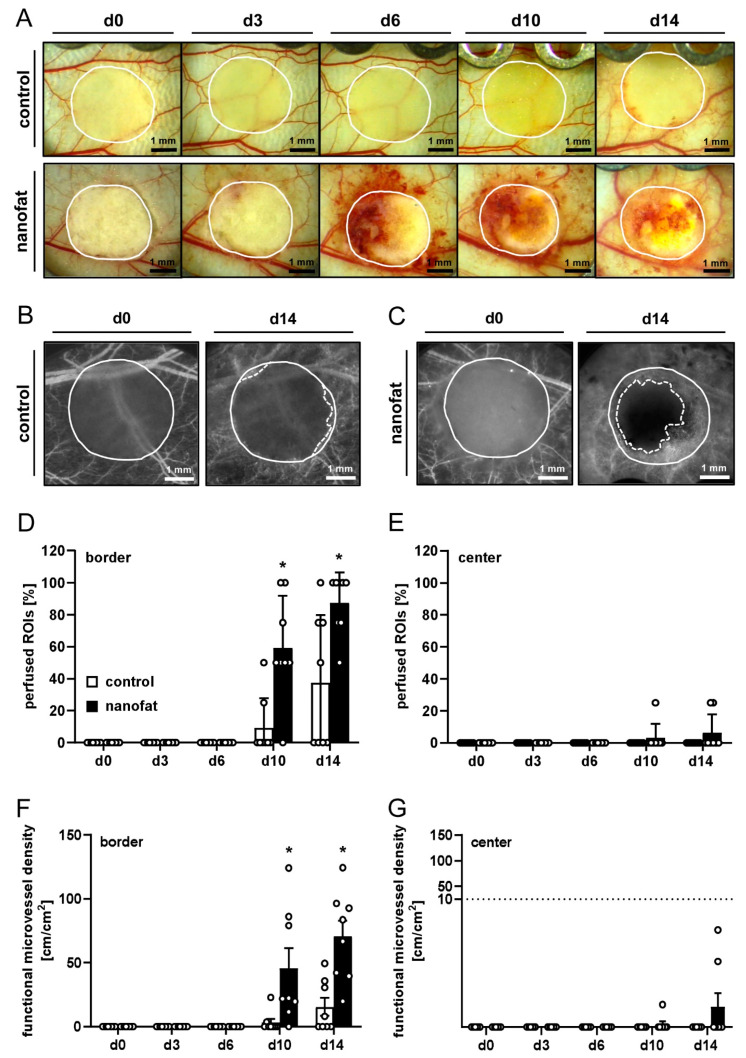
In vivo microscopy of dermal substitutes. (**A**) Stereomicroscopy of non-seeded (control, upper panels) and nanofat-seeded (lower panels) dermal substitutes on days 0, 3, 6, 10 and 14 (implant border = closed line). (**B**,**C**) Intravital fluorescence microscopy of non-seeded (control, (**B**)) and nanofat-seeded (**C**) dermal substitutes on days 0 and 14 (implant border = closed line; border of non-vascularized implant area = broken line). (**D**–**G**) Perfused regions of interest (ROIs) (%) (**D**,**E**) and functional microvessel density (cm/cm^2^) (**F**,**G**) in the border (**D**,**F**) and center zones (**E**,**G**) of non-seeded (control; white bars, *n* = 8) and nanofat-seeded (black bars, *n* = 8) dermal substitutes on days 0, 3, 6, 10 and 14 after implantation, as assessed by intravital fluorescence microscopy. Means ± standard errors of the mean (SEMs). * *p* < 0.05 vs. control.

Stereomicroscopic imaging showed a different reaction of the host tissue to nanofat-seeded dermal substitutes when compared to non-seeded controls. Of note, the nanofat-seeded implants induced a strong angiogenic response, as indicated by the formation of hemorrhages around the implants between days 6 and 14 (Figure 2A).

Repeated intravital fluorescence microscopy of the implants demonstrated that nanofat-seeded dermal substitutes induced the development of dense microvascular networks in their border zones and also the ingrowth of a few microvessels into their center until the end of the 14-day observation period. In contrast, non-seeded dermal substitutes did not show significant vessel ingrowth (Figure 2B,C). In line with these observations, we found a significantly higher number of perfused ROIs and functional microvessel density in the border zones of nanofat-seeded dermal substitutes on days 10 and 14 when compared to controls (Figure 2D–G). Additional measurements of microhemodynamic parameters showed that the centerline RBC velocity, shear rate and volumetric blood flow of individual microvessels were markedly higher in the group of nanofat-seeded dermal substitutes when compared to the control group (Table 1).

**Figure 3:**
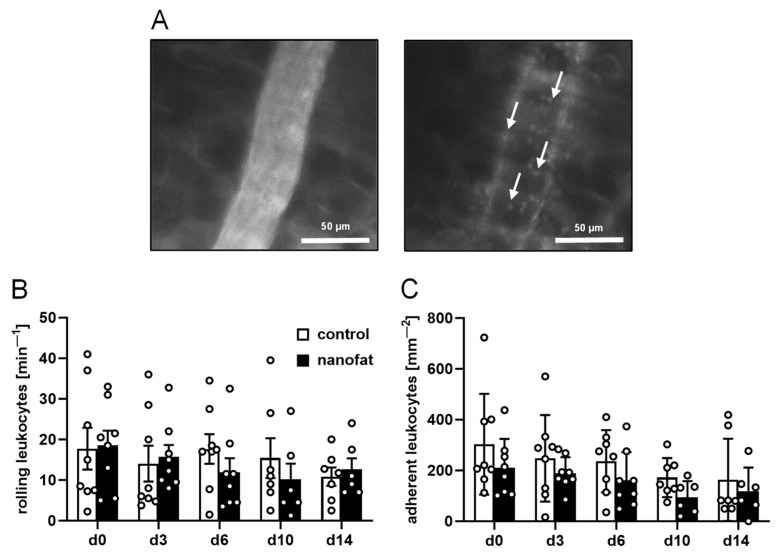
Leukocyte–endothelial cell interactions in response to dermal substitutes. (**A**) Intravital fluorescence microscopy of a collecting venule next to a dermal substitute (blue light epi-illumination, contrast enhancement by 5% fluorescein isothiocyanate (FITC)-labeled dextran (left panel); green light epi-illumination, in situ staining of leukocytes with 0.1% rhodamine 6G (right panel); arrows = leukocytes). (**B**,**C**) Rolling leukocytes (min^−1^) (**B**) and adherent leukocytes (mm^−2^) (**C**) within postcapillary and collecting venules in direct vicinity to non-seeded (control; white bars, *n* = 8) and nanofat-seeded (black bars, *n* = 8) dermal substitutes throughout the 14-day observation period. Means ± SEMs.

To assess the inflammatory host tissue response to the implants, leukocyte–endothelial cell interactions were assessed over time in postcapillary and collecting venules in the direct vicinity of the implants. Of note, in both groups, these vessels exhibited comparable diameters, centerline RBC velocities, shear rates and volumetric blood flows, indicating standardized microhemodynamic conditions (Table 2). Moreover, there were no statistically significant differences in the numbers of rolling and adherent leukocytes throughout the observation period (Figure 3A–C).

### 3.2. Histological and Immunohistochemical Analysis of the Dermal Substitutes

**Figure 4:**
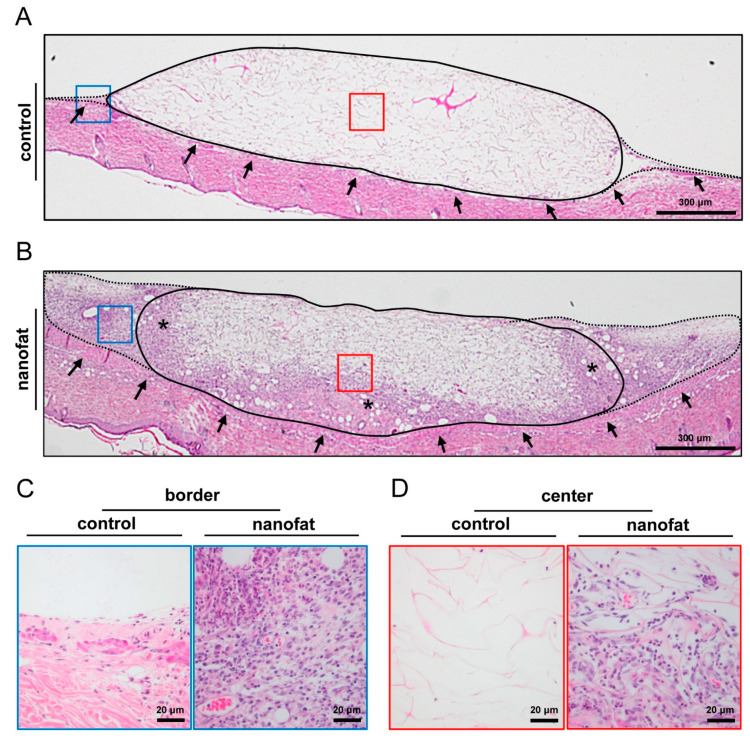
Tissue integration of dermal substitutes. (**A**,**B**) HE-stained sections of non-seeded (control, (**A**)) and nanofat-seeded (**B**) dermal substitutes on day 14 after implantation within dorsal skinfold chambers of C57BL/6J recipient mice (implant border = closed line; border zone = broken line; ROIs in the border and center zones of the implants shown in higher magnification in C and D = blue and red frame; panniculus carnosus muscle = arrows; granulation tissue = asterisks). (**C**,**D**) Higher magnification of blue and red frames in A and B in the border (**C**) and center (**D**) zones of the implants.

At the end of the in vivo experiments, on day 14, the implants were additionally analyzed by means of histology and immunohistochemistry. Nanofat-seeded dermal substitutes exhibited an improved tissue integration, with the formation of a dense, vascularized granulation tissue surrounding the implants when compared to non-seeded controls (Figure 4A–C). Notably, this granulation tissue also progressively grew into the pores of nanofat-seeded dermal substitutes, filling up substantial areas of the implants on day 14 (Figure 4A,B,D).

**Figure 5:**
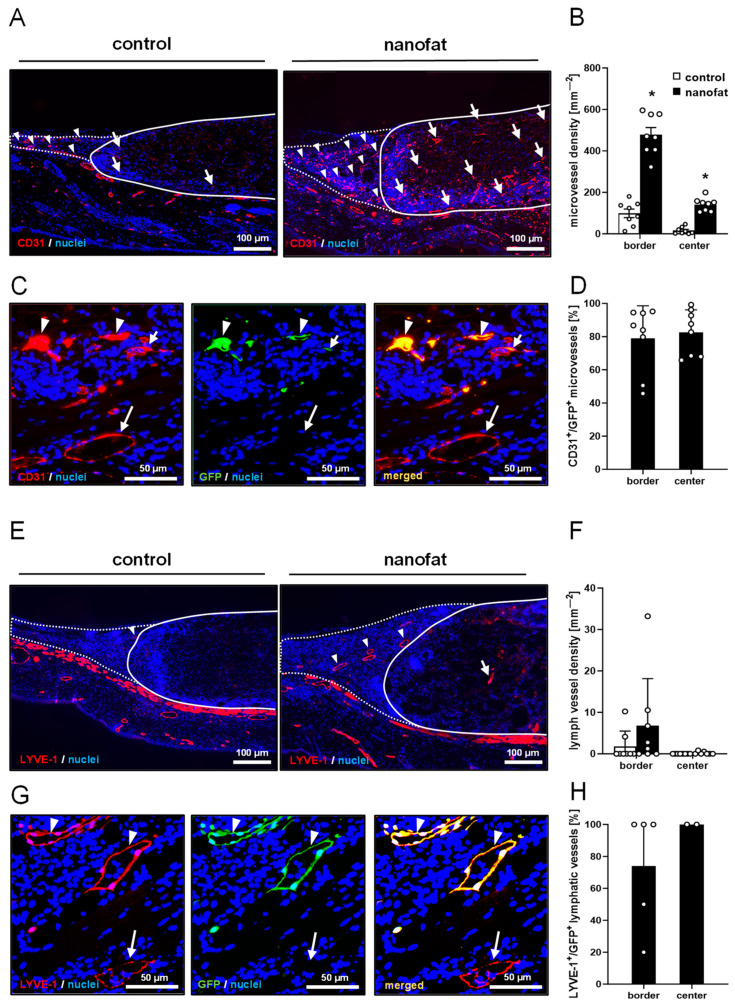
Vascularization and lymphatic drainage of dermal substitutes. (**A**) Immunohistochemical detection of CD31^+^ microvessels in the border zones (arrowheads) and the center (arrows) of non-seeded (control) and nanofat-seeded dermal substitutes on day 14 (implant border = closed line; border zones = dotted line). (**B**) Microvessel density (mm^−2^) of non-seeded (control; white bars, *n* = 8) and nanofat-seeded (black bars, *n* = 8) dermal substitutes on day 14, as assessed by immunohistochemistry. Means ± SEMs. * *p* < 0.05 vs. control. (**C**) Immunohistochemical detection of CD31^+^/GFP^−^ (arrows) and CD31^+^/GFP^+^ (arrowheads) microvessels in nanofat-seeded dermal substitutes on day 14. (**D**) CD31^+^/GFP^+^ microvessels (%) in the border zones and the center of nanofat-seeded dermal substitutes on day 14, as assessed by immunohistochemistry. Means ± SEMs. (**E**) Immunohistochemical detection of lymphatic vessel endothelial hyaluronan receptor (LYVE)-1^+^ lymph vessels in the border zones (arrowheads) and the center (arrow) of non-seeded (control) and nanofat-seeded dermal substitutes on day 14 (implant border = closed line; border zones = dotted line). (**F**) Lymph vessel density (mm^−2^) of non-seeded (control; white bars, *n* = 8) and nanofat-seeded (black bars, *n* = 8) dermal substitutes on day 14, as assessed by immunohistochemistry. Means ± SEMs. (**G**) Immunohistochemical detection of LYVE-1^+^/GFP^-^ (arrow) and LYVE-1^+^/GFP^+^ (arrowheads) lymph vessels in nanofat-seeded dermal substitutes on day 14. (**H**) LYVE-1^+^/GFP^+^ microvessels (%) in the border zones and the center of nanofat-seeded dermal substitutes on day 14, as assessed by immunohistochemistry. Means ± SEMs.

The immunohistochemical detection of the endothelial cell marker CD31 showed that nanofat-seeded dermal substitutes exhibited a 4.6-fold higher and 9-fold higher microvessel density in the border and center zones, respectively, when compared to controls (Figure 5A,B). Of interest, GFP/CD31 co-staining revealed that ~80% of the microvessels within the nanofat-seeded implants were GFP^+^, indicating their origin from the seeded GFP^+^ nanofat (Figure 5C,D). In addition, immunohistochemical detection of LYVE-1 showed that there were a few lymphatic vessels in the newly developing granulation tissue surrounding the implants without significant differences in the lymph vessel density between the two groups (Figure 5E,F). However, in contrast to the control group, such LYVE-1^+^ lymph vessels could also be detected in the center of two nanofat-seeded dermal substitutes (Figure 5E,F). Again, most of these lymph vessels were GFP^+^ (Figure 5G,H).

**Figure 6:**
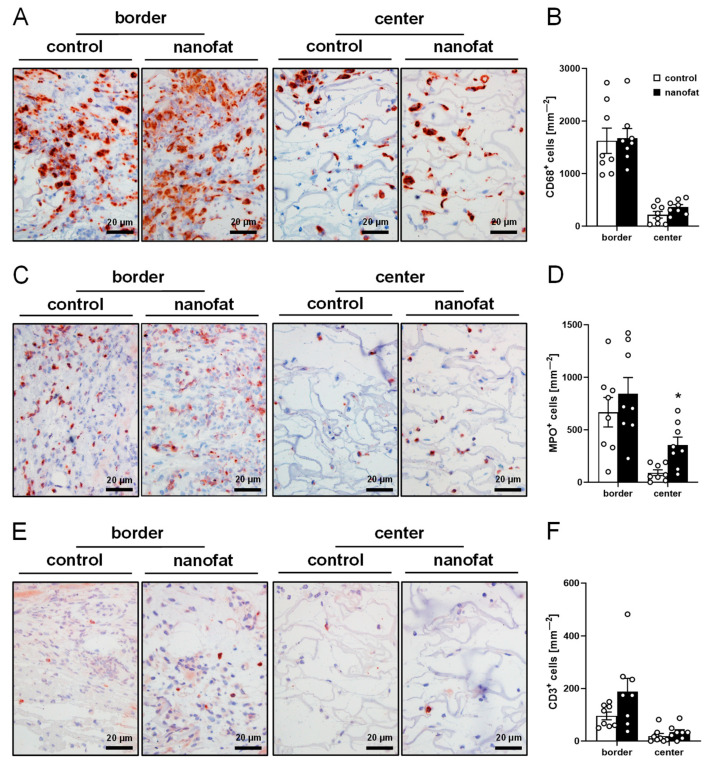
Immune cell infiltration into dermal substitutes. (**A**,**C**,**E**) Immunohistochemical detection of CD68^+^ macrophages (**A**), myeloperoxidase (MPO)^+^ granulocytes (**C**) and CD3^+^ lymphocytes (**E**) in the border zones and the center of non-seeded (control) and nanofat-seeded dermal substitutes on day 14. (**B**,**D**,**F**) CD68^+^ macrophages (mm^−2^) (**B**), MPO^+^ granulocytes (mm^−2^) (**D**) and CD3^+^ lymphocytes (mm^−2^) (**F**) in the border zones and the center of non-seeded (control; white bars, *n* = 8) and nanofat-seeded (black bars, *n* = 8) dermal substitutes on day 14, as assessed by immunohistochemistry. Means ± SEMs. * *p* < 0.05 vs. control.

**Figure 7:**
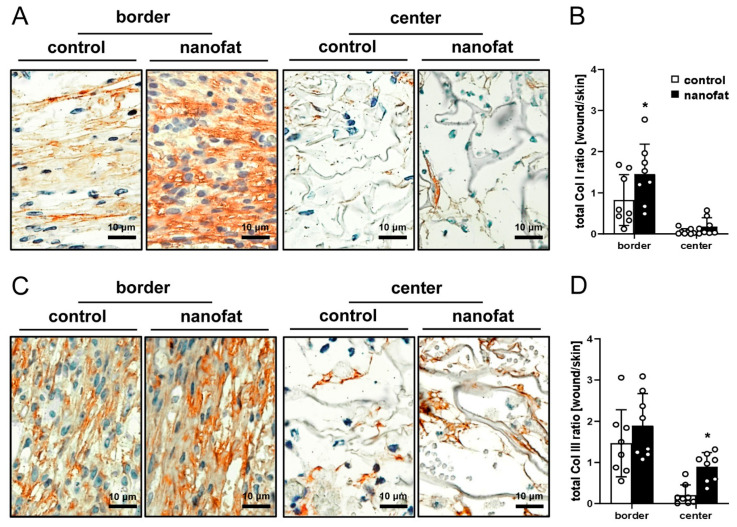
Collagen contents of dermal substitutes. (**A**,**C**) Immunohistochemical detection of collagen (Col) I (**A**) and III (**C**) in the border zones and the center of non-seeded (control) and nanofat-seeded dermal substitutes on day 14. (**B**,**D**) Total Col I (**B**) and Col III (**D**) ratio (implant/skin) in the border zones and the center of non-seeded (control; white bars, *n* = 8) and nanofat-seeded (black bars, *n* = 8) dermal substitutes on day 14, as assessed by immunohistochemistry. Means ± SEMs. * *p* < 0.05 vs. control.

To assess the immune cell infiltration of the implants, histological sections were stained with antibodies against CD68 (macrophages), MPO (neutrophilic granulocytes) and CD3 (lymphocytes). The quantitative analysis of these staining experiments did not show marked differences between nanofat-seeded and non-seeded dermal substitutes (Figure 6A–F). There was, however, a clear difference in the collagen content of both implant types. In fact, nanofat-seeded dermal substitutes exhibited a significantly higher total Col I ratio in the border zones and total Col III ratio in the center when compared to controls (Figure 7A–D).

## 4. Discussion

Dermal substitutes, such as Integra^®^, provide a sound solution for the primary treatment of large, full-thickness skin defects. For the final coverage with STSGs, a sufficient vascularization of these substitutes is of major importance. To introduce an approach that could be implemented easily into daily clinical practice, we analyzed in this preclinical study the effects of nanofat on implanted dermal substitutes. Of interest, we found that their seeding with freshly generated nanofat markedly improves their vascularization and integration into the surrounding host tissue without affecting their biocompatibility.

Previous studies have already demonstrated that the biological activation of dermal substitutes with angiogenic growth factors, such as basic fibroblast growth factor (bFGF) as well as isolated stem cells or MVFs, is effective in stimulating the formation of new microvascular networks inside their matrix structure after implantation [9,18,19,20]. The herein-used nanofat bears the main advantage that it contains all these individual components, which may synergistically promote implant vascularization and tissue integration. For instance, in a recent study, we reported that the immunohistochemically assessed density of MVFs in murine nanofat is ~300–400 mm^−2^ [21]. Moreover, nanofat can be rapidly generated intraoperatively by mechanical emulsification of fat samples without the need for laborious biofabrication techniques. Finally, it exhibits a liquid consistency, which is beneficial for the seeding of biomaterials [22,23]. Accordingly, we could easily seed dermal substitutes of a standardized size by incubating them in freshly generated nanofat. Histological analyses revealed that this resulted in the sticking of nanofat within the surface pores of the samples.

For in vivo analyses, nanofat-seeded and non-seeded dermal substitutes were implanted into well-defined, full-thickness skin defects within dorsal skinfold chambers of recipient mice. This approach allowed the repeated imaging of the implants through the observation window of the chambers throughout a time period of 14 days, while protecting them from exsiccation or manipulation by the animals [24]. Using intravital fluorescence microscopy, it was possible to study the formation of new microvessels inside the implants and to assess the functionality and microhemodynamics of individual blood vessels by direct visualization of blood perfusion.

As already shown in previous experimental studies [9], non-seeded Integra^®^ exhibited a slow vascularization and modest tissue integration. This problem could be solved by seeding the dermal substitute with nanofat. This modification resulted in a significantly higher number of perfused ROIs and functional microvessel density in the border zones of the implants on days 10 and 14 when compared with non-seeded controls. In line with these findings, we also detected an improved blood perfusion of nanofat-seeded dermal substitutes with a significantly higher centerline RBC velocity, shear rate and volumetric blood flow in individual microvessels. However, intravital fluorescence microscopy in the epi-illumination technique only allowed the assessment of these microhemodynamic parameters in microvessels located on the surface of the implants. To overcome this limitation, we additionally analyzed the vascularization and tissue integration of the implants on immunohistochemically stained cross-sections at the end of the 14-day observation period. These analyses showed that nanofat-seeded dermal substitutes exhibited a markedly higher density of CD31^+^ microvessels in both their border zones and center when compared to controls.

The beneficial effects of nanofat on the vascularization of implanted dermal substitutes may be best explained by the interplay of several biological mechanisms. Nanofat contains many fully functional microvessel segments, which are able to rapidly reassemble into new microvascular networks and develop interconnections with the surrounding host vessels via inosculation [9,15,25]. Hence, we found that ~80% of all microvessels detected in both the center and border zones of the implants originated from microvessel segments of the seeded GFP^+^ nanofat. In addition, nanofat releases substantial amounts of pro-angiogenic growth factors [14,23], which may have stimulated the stepwise ingrowth of new microvessels from the surrounding tissue into the implants. Accordingly, the center and border zones of nanofat-seeded dermal substitutes contained a fraction of ~20% GFP^-^ microvessels originating from the host tissue. Finally, it should be mentioned that nanofat is also a rich source of ADSCs, which can differentiate into various cell lines, including endothelial cells and vascular smooth muscle cells [26,27]. For instance, Cohen et al. [28] reported that depending on the processing technique, the stromal vascular fraction of nanofat contains ~28–37% of ADSCs. Therefore, these cells may have also contributed to the formation of new blood vessels within the implants.

In addition to the development of new microvessels, a sufficient lymphatic drainage has been proposed to be beneficial for the adequate tissue integration of skin grafts and dermal substitutes, because it reduces edema formation and promotes the removal of local debris [29]. In this context, we recently showed that nanofat generated from the subcutaneous adipose tissue of donor mice not only contains microvessel segments but also lymphatic vessel fragments [30]. In line with these findings, we herein also detected LYVE-1^+^/GFP^+^ lymph vessels in nanofat-seeded dermal substitutes. However, this observation was rare, so their presence may have not exerted significant effects on the integration of the dermal substitutes during the first 14 days after implantation.

For their safe use in patients, biomaterials should not induce a strong inflammatory host tissue reaction. On the other hand, it is well known that a mild inflammation can promote the rapid vascularization of biomaterial implants [31]. In fact, there is a close connection between inflammation and angiogenesis, largely because immune cells produce various pro-angiogenic factors [32]. Accordingly, fundamental regenerative processes, such as wound healing, also typically involve an early inflammatory phase that is followed by a phase of blood vessel development [33]. Against this background, we herein additionally analyzed the leukocyte–endothelial cell interaction in postcapillary and collecting venules close to the implants and the final immune cell infiltration of the dermal substitutes. However, no marked differences between the groups of nanofat-seeded and non-seeded dermal substitutes were observed, leading to two conclusions: Firstly, nanofat does not affect the inflammatory host tissue response to implanted Integra^®^ and, thus, its good biocompatibility. Secondly, inflammation is not the primary driving force responsible for the improved vascularization of nanofat-seeded dermal substitutes.

Finally, we performed an immunohistochemical analysis of collagen formation in the border zones and center of the implants. Of interest, we found that nanofat-seeded dermal substitutes exhibited a significantly higher total Col I ratio in the border zones and total Col III ratio in the center when compared to controls. From wound healing studies, it is well known that newly forming granulation tissue exhibits a high content of Col III, whereas tissue maturation and remodeling in later stages of the wound healing process are associated with a shift toward increased Col I levels [34,35]. These findings further support the conclusion that nanofat not only improves but also accelerates tissue integration of dermal substitutes in full-thickness skin defects. In fact, this integration started with the formation of a granulation tissue surrounding the implants. After 14 days, this granulation tissue exhibited an advanced maturation stage in the group of nanofat-seeded dermal substitutes, as indicated by a higher Col I content when compared to controls. In addition, more immature granulation tissue had already invaded central areas of the nanofat-seeded implants at this time point, resulting in a higher Col III content in the center.

It should be mentioned that the present study also exhibits some limitations. To provide a proof of principle, we only compared nanofat-seeded implants and non-seeded controls without testing alternative vascularization strategies in the identical setting. Moreover, the vascularization and tissue integration of the dermal substitutes were only analyzed during the first 14 days after implantation. A technical reason for this is the fact that the dorsal skinfold chambers should not be used for longer on the backs of the mice. In fact, the elasticity of the dorsal skinfold changes over time, which may result in the tilting of the chamber frames and, thus, perfusion failure of the chamber tissue at later observation time points. However, the major aim of our study was to analyze whether nanofat can accelerate the vascularization of implanted dermal substitutes. Hence, to respond to this question, the initial 14 days after implantation formed the ideal observation period.

## 5. Conclusions

The present study demonstrates for the first time that nanofat seeding improves the vascularization and integration of dermal substitutes without affecting their biocompatibility. The fact that the use of nanofat is already well-established in reconstructive surgery makes this approach attractive for the treatment of full-thickness skin defects, eventually reducing the time period needed for final skin reconstruction and, thus, the patients’ dependence on sanitary infrastructure, such as outpatient-based wound care and/or inpatient treatment. Beyond this specific application, it can be assumed that nanofat may also be suitable for improving the in vivo performance of other biomaterials, such as surgical meshes for hernia repair or breast reconstruction as well as implanted bone substitutes. However, as is the case for other autologous products, such as platelet-rich plasma or chimeric antigen receptor (CAR) T-cells [36,37], the composition and biological activity of nanofat can significantly vary between individual donors. Accordingly, more systematic studies will be required to investigate the efficiency of using nanofat for the aforementioned clinical applications in consideration of individual patient-specific characteristics, which may finally result in the broad implementation of this fat derivative into personalized treatment strategies.

## Figures and Tables

**Table 1:** Diameter (µm), centerline red blood cell (RBC) velocity (µm/s), shear rate (s^−1^) and volumetric blood flow (pL/s) of microvessels within the border and center zones of non-seeded (control; *n* = 8) and nanofat-seeded (*n* = 8) dermal substitutes. Means ± SEMs; * *p* < 0.05 vs. control.

	d0	d3	d6	d10	d14
***diameter (µm):***					
border: control	-	-	-	13.9 ± 4.6	13.5 ± 1.1
nanofat	-	-	-	17.1 ± 0.7	15.7 ± 0.5
center: control	-	-	-	-	-
nanofat	-	-	-	-	20.9 ± 4.6
***centerline RBC velocity (µm/s):***					
border: control	-	-	-	57.4 ± 26.4	53.1 ± 17.6
nanofat	-	-	-	217.4 ± 14.2 *	205.7 ± 21.9 *
center: control	-	-	-	-	-
nanofat	-	-	-	-	131.8 ± 113.0
***shear rate (s^−1^):***					
border: control	-	-	-	47.7 ± 34.3	32.2 ± 12.3
nanofat	-	-	-	105.9 ± 8.6 *	95.4 ± 7.8 *
center: control	-	-	-	-	-
nanofat	-	-	-	-	63.8 ± 57.5
***volumetric blood flow (pL/s):***					
border: control	-	-	-	8.0 ± 4.4	6.3 ± 2.8
nanofat	-	-	-	33.1 ± 3.0 *	29.2 ± 6.7 *
center: control	-	-	-	-	-
nanofat	-	-	-	-	21.7 ± 15.5

**Table 2:** Diameter (µm), centerline RBC velocity (µm/s), shear rate (s^−1^) and volumetric blood flow (pL/s) of postcapillary and collecting venules in direct vicinity to non-seeded (control; *n* = 8) and nanofat-seeded (*n* = 8) dermal substitutes. Means ± SEMs.

	d0	d3	d6	d10	d14
***diameter (µm):***					
control	35.8 ± 0.9	37.9 ± 1.7	36.5 ± 2.6	40.8 ± 2.5	33.5 ± 1.6
nanofat	39.5 ± 2.1	38.3 ± 1.3	37.9 ± 1.4	34.7 ± 1.4	31.5 ± 1.8
***centerline RBC velocity (µm/s):***					
control	592.7 ± 84.0	667.3 ± 223.1	604.1 ± 111.5	491.7 ± 124.4	343.4 ± 80.6
nanofat	489.1 ± 54.6	543.2 ± 56.4	691.6 ± 74.0	391.7 ± 62.3	346.0 ± 110.0
***shear rate (s^−1^):***					
control	133.2 ± 17.3	138.4 ± 21.7	129.6 ± 21.3	103.9 ± 31.3	81.3 ± 17.5
nanofat	98.1 ± 7.7	105.1 ± 8.4	150.7 ± 18.2	89.4 ± 11.4.	84.4 ± 24.3
***volumetric blood flow (pL/s):***					
control	382.5 ± 62.0	521.6 ± 102.7	461.1 ± 112.6	407.6 ± 83.9	216.6 ± 62.1
nanofat	430.5 ± 102.3	439.8 ± 77.1	575.4 ± 71.2	256.9 ± 53.6	196.9 ± 76.0

## Data Availability

The original contributions presented in this study are included in the article and further inquiries can be directed to the corresponding author.
